# Aging and obesity are associated with undiagnosed hypertension in a cohort of males in the Central Province of Sri Lanka: a cross-sectional descriptive study

**DOI:** 10.1186/s12872-017-0600-8

**Published:** 2017-06-21

**Authors:** N. W. I. A. Jayawardana, W. A. T. A. Jayalath, W. M. T. Madhujith, U. Ralapanawa, R. S. Jayasekera, S. A. S. B. Alagiyawanna, A. M. K. R. Bandara, N. S. Kalupahana

**Affiliations:** 1 Department of Animal and Food Sciences, Faculty of Agriculture, Rajarata University of Sri Lanka, Anuradhapura, Sri Lanka; 2 Department of Medicine, Faculty of Medicine, University of Peradeniya, Peradeniya, Sri Lanka; 3 Department of Food Science and Technology, Faculty of Agriculture, University of Peradeniya, Peradeniya, Sri Lanka; 4 National Transport Medical Institute, Kandy, Sri Lanka; 5 Department of Agricultural Systems, Faculty of Agriculture, Rajarata University of Sri Lanka, Anuradhapura, Sri Lanka; 6 Department of Physiology, Faculty of Medicine, University of Peradeniya, Peradeniya, Sri Lanka

**Keywords:** Hypertension, Age, BMI, Obesity, Lifestyle factors, Systolic and diastolic blood pressure, South Asians

## Abstract

**Background:**

Lifestyle factors associated with hypertension (HT) in South Asian populations are relatively unknown. The objective of the current study was to investigate the prevalence rates of undiagnosed HT and factors associated with it in a cohort of males from the Central Province of Sri Lanka.

**Methods:**

The study group consisted of 2462 males (mean age 31 years, range: 16–72 years) who underwent a routine medical examination at the National Transport Medical Institute, Kandy, Sri Lanka. Participants with previously diagnosed heart disease, diabetes, hypertension or other chronic illnesses were excluded from this study. Dietary and other lifestyle factors were studied using validated self-administered questionnaires. Body Mass Index (BMI) cut-off values for Asians were used to categorize the subjects according to weight status. The association of individual dietary and lifestyle patterns with HT was assessed by fitting into binary logistic regression models.

**Results:**

The mean systolic (SBP) and diastolic blood pressures (DBP) of the individuals were 125.9 mmHg and 80.5 mmHg, respectively. The prevalence rate of undiagnosed HT was 31.7%. Both SBP and DBP showed significant positive correlations with age, weight, BMI and waist circumference. After adjusting for potential confounders, HT was associated with older age (*p* = 0.001) and increased weight status (*p* < 0.001) with trends of association for level of education (*p* = 0.058). Level of income, alcohol consumption, sleeping hours, smoking, physical activity level, ethnic difference, consumption of fruits, fish, meat, dairy, sweets or fried snacks were not significantly associated with HT. Obese males were 92.1% [odds ratio: 1.9 (1.4–2.7)] more likely to be hypertensive compared to normal weight males.

**Conclusions:**

Undiagnosed HT is prevalent at an alarming rate among adult males in the Central Province of Sri Lanka. Its association with age and BMI (weight status) highlights the importance of routine screening for HT as well as interventions targeted at reducing obesity to curb the rise of this modifiable cardiovascular disease risk factor.

## Background

An estimated 800 million adults in the world are suffering from hypertension (HT) [1], a cardiovascular disease risk factor which is estimated to account for 6% of all deaths [2]. The prevelance of HT has also increased steadily over the last two decades [1]. Around 80% of the burden of hypertension and its complications are observed in low and middle-income countries [3]. Economic development, industrialization, nutrition transition and globalization that lead to a rapidly change in lifestyles could at least in part account for the increasing prevalence of hypertension. More than a quarter of the world’s population is already hypertensive and this number is projected to increase up to 29% by 2025 [4]. However, hypertension can be asymptomatic [5] and many people with hypertension may not get any treatment until the outcomes become serious. The detection and control of HT is thus a major public health challenge. Sri Lanka is a South Asian nation with lower-middle income status. According to the World Health Organization (WHO) non-communicable diseases country profiles, the prevalence of hypertension among Sri Lankans was 39.2% (41.4% in males and 37.1% in females) in year 2008 [6].

While the relationship between lifestyle patterns and hypertension has been extensively studied in western populations, less data is available regarding the risk factors associated with hypertension in South Asia. Since the dietary habits and physical activity patterns of Sri Lankans are different from that of western counterparts [7], it is imperative to find out the exact risk factors for HT in Sri Lanka, so that effective interventions can be tailored to prevent this condition.

To date, the only national study conducted in Sri Lanka by Katulanda et al., has shown that male gender is a significant risk factor for developing hypertension [8]. Thus, the aim of the present study was to determine the underlying lifestyle factors associated with hypertension among a cohort of males in the Central Province of Sri Lanka.

## Methods

### Research design and population

The data for this study were obtained from the baseline assessment of the National Transport Medical Test Cohort, a prospective study being conducted to assess lifestyle factors associated with non-communicable disease risk factors in the Central Province of Sri Lanka. Detailed methods have been previously published [9]. Briefly, the current analysis was a cross-sectional descriptive study in 2462 adult males (age range 16 to 72 years), who presented for a routine medical evaluation at the National Transport Medical Institute, Kandy, Sri Lanka from January 2013 to February 2014. Participants with previously diagnosed heart diseases, diabetes, hypertension or other chronic illnesses were excluded from this study.

### Ethics, consent and permissions

This study was approved by the ethics review committee (Institutional review board) of the Faculty of Medicine, University of Peradeniya, Sri Lanka (2015/EC/13). Informed written consent was obtained from all participants.

### Data collection

#### Systolic and diastolic blood pressure

Two readings of systolic and diastolic blood pressure were taken from the right arm of each subject in a sitting position after a 10-min rest using a standard clinical mercury manometer (0125B Accoson Dekamet Mercury Sphygmomanometer, UK). Mean value of the two readings were taken as the BP of the participant. A systolic blood pressure (SBP) of ≥140 mmHg or diastolic blood pressure (DBP) of ≥90 mmHg were considered as the cut-off level to determine the presence of hypertension [10].

#### Anthropometric measurements

WHO guidelines were followed in obtaining height, weight and waist circumference (WC) measures [11]. Height was measured using a stadiometer (Healthweigh® Mechanical Physician Scale (RL-MPS), Goldbell Weigh-System, Singapore) to the nearest millimetre. Weight was measured using a digital scale (Healthweigh® Mechanical Physician Scale (RL-MPS), Goldbell Weigh-System, Singapore) to the nearest 100 g. An inelastic measuring tape was used to measure the waist circumference to the nearest millimetre at the midpoint between the lowest palpable rib and the superior border of the iliac crest in the mid axillary line at the end of normal expiration.

The body mass index (BMI) was calculated as weight (kg) / height^2^ (m^2^). Weight status was categorized using the BMI cutoff values for Asians as defined by WHO [12]. The weight categories were: underweight (BMI <18.5 kg/m^2^), normal weight (BMI 18.5–22.9 kg/m^2^), overweight (23–27.5 kg/m^2^ and obese (> 27.5 kg/m^2^). WC ≥90 cm was defined as abdominal obesity [13].

#### Dietary, physical activity and other lifestyle data

A validated self-administered food frequency questionnaire was used to collect dietary data reflecting the consumption over the past six months. The short version of the International Physical Activity Questionnaire (IPAQ) was employed to assess the physical activity during one week [14]. Details of the questionnaires have been previously published [9]. Alcohol consumption, smoking and sleep duration were collected using a self-administered questionnaire. Physical activity levels were categorized into low (< 150 min of moderate-intensity physical activity or 75 min of vigorous-intensity activity per week), medium (150–300 min of moderate-intensity activity or 75–150 min of vigorous-intensity physical activity per week) or high (> 300 min of moderate-intensity physical activity per week) [14–16]. Regarding the smoking score, moderate smokers were defined as a person smoking 1–10 cigarettes per day, while heavy smokers were defined as smoking >10 cigarettes per day [17]. When sleep hours were considered, a low sleep score was given to individuals who slept for <6 h per day while medium and high scores were given for persons who slept for 6–8 h per day and >8 h per day respectively [18]. Demographic data on age, ethnicity, level of education and household income were collected by interviewer-administrated questionnaires.

#### Statistical analysis

Data analysis was performed without imputing for missing values. Mean and Standard Deviation (SD) were calculated for continuous variables while frequencies and percentages were calculated for categorical variables. The mean differences of SBP and DBP between age categories, ethnic groups and educational levels were tested for significance by fitting a ANOVA model for each variable separately and mean separation was done using Duncan’s new multiple range test (DNMRT). Correlation between variables was analysed for significance with the Pearson correlation coefficient. Two binary logistic regression models were fitted to assess the associations of individual dietary patterns and other lifestyle patterns with hypertension. In these models, healthy (non-hypertensive) group was used as the reference category and the effect of each variable was tested after adjusting for other confounding variables. Further, in logistic regression analysis, independent variables; alcohol intake, sleeping hours, smoking, consumption of fruits, fish, meat, dairy, sweets and fried snacks were considered as numerical variables and entered into the model as frequency per week; and age group, education level, income level, weight status and ethnicity were entered into the model as categorical variables in model 1 and education level was considered as an ordinal variable in model 2. All analysis was carried out using SAS 9.3 (SAS Institute Inc., Cary, NC) considering a significance level of 0.05.

## Results

Of the 2462 subjects, 1627 (66.1%) were less than 35 years old, while 566 (22.9%), 227 (9.2%) and 42 (1.7%) belonged to 35–44, 45–54 and 55 years or above categories respectively. The majority of the sample were Sinhalese (2065–84.1%), followed by Moors (201–8.2%) and Tamils (191–7.8%). Other baseline characteristics of the sample are summarized in Table 1. Eighty-four percent of the study subjects had an education level of Ordinary Level or above, while 85% had a monthly income in the range of LKR 13,000 to 50,000 (US $1 ~ LKR 150). The prevalence rate of previously undiagnosed hypertension of the study group was 31.7%. The prevalence rates of overweight and obesity in the sample were 31.8% and 12.3%, respectively. Moreover, 17.1% of the subjects were centrally obese.

**Table 1:** Baseline characteristics of the study sample

Characteristic	Mean	± SD
Age (years)	31	10.3
Height (m)	1.6	0.1
Weight (kg)	62.5	11.9
Systolic blood pressure (mm Hg)	125.9	11.2
Diastolic blood pressure (mm Hg)	80.5	7.3
BMI (kg m^−2^)	22.7	4.2
WC (cm)	78.6	11.3

*SBP* Systolic blood pressure, *DBP* Diastolic blood pressure, *BMI* body mass index, *SD* standard deviation, *WC* waist circumference

When the lifestyle patterns were considered, 22.5% of the study group consumed alcohol and 14.4% were smokers (14.2% of them were moderate smokers and 0.2% were heavy smokers). While 78.6% of participants reported having 7–8 h of sleep per day, 64.5% of the participants indicated that they engage in 300 min or more of moderate-intensity physical activity per week. The study group had a relatively low fruit intake with only 14.5% of participants eating more than 1 fruit per day. Detailed description of the demographics and lifestyle habits of these participants have been recently published elsewhere [9].

To study the different factors associated with HT in this group, first we compared the mean SBP and DBP levels between different groups according to age, ethnicity, education level and physical activity level (Table 2). The mean SBP and DBP showed a significant difference between age groups, with a trend towards increasing blood pressure with aging. No significant difference was observed in mean SBP and DBP among ethnic groups. While participants who had educated up to grade 5 or less had significantly higher mean SBP compared to other educational levels, this was a modest difference with no difference of DBP according to educational status (Table 2). Finally, there was no difference in mean SBP or DBP among the different levels of self-reported physical activity levels.

**Table 2:** Mean systolic and diastolic blood pressures according to age, ethnicity, education level and physical activity level

Category	Mean SBP (±SD), mm Hg	Mean DBP (±SD), mm Hg
Age category (years) (*n* = 2462)		
< 20	124.1^a^ (10.6)	78.9^a^ (6.9)
20–30	124.8^a,b^ (10.8)	79.9^a,b^ (7.2)
31–40	126.0^b^ (10.8)	80.7^b^ (7.2)
41–50	128.4^c^ (12.4)	82.4^c^ (7.3)
> 50	130.7^d^ (11.4)	82.9^c^ (8.5)
Ethnicity (*n* = 2457)		
Sinhalese	125.9^a^ (11.3)	80.5^a^ (7.4)
Tamil	126.1^a^ (9.3)	80.8^a^ (6.1)
Moor	126.0^a^ (12.2)	80.7^a^ (8.6)
Education level (*n* = 2339)		
No education – grade 5	131.2^a^ (11.1)	82.6^a^ (5.6)
Grade 6 – grade 11	126.1^b^ (11.8)	80.4^a^ (7.6)
Ordinary level passed	125.9^b^ (10.8)	80.8^a^ (7.3)
Advanced level passed	125.5^b^ (11.5)	80.1^a^ (7.3)
Graduate/postgraduate	129.4^a,b^ (13.1)	81.7^a^ (6.8)
Physical activity level (*n* = 2462)		
Low	125.7^a^ (11.6)	80.3^a^ (7.6)
Medium	125.9^a^ (10.7)	80.2^a^ (7.3)
High	126.1^a^ (11.0)	80.7^a^ (7.2)

^a,b,c,d^ Mean SBP and DBP values with different superscripts in a column within the same category are significantly different (*p* < 0.05)

Next we studied the associations between age, indices of adiposity and lifestyle factors with SBP and DBP. Similar to the findings of increased blood pressure in different age groups, we found a significant positive correlation of both SBP and DBP with age (Table 3). Moreover, indices of adiposity (BMI and WC) showed significant positive correlations with both SBP and DBP. In contrast, there were no significant correlations between intensity of physical activities (high, medium, low), sleep or smoking scores with SBP or DBP.

**Table 3:** Correlation (linear) of systolic and diastolic blood pressure with age, smoking score, sleeping core, physical activity level, BMI, waist circumference and weight

	Correlation co-efficient (*r*)	
	SBP	DBP
Age	0.166*	0.163*
Smoking score	−0.008	−0.001
Sleep score	−0.010	0.006
BMI	0.166*	0.192*
WC	0.200*	0.216*
Weight	0.168*	0.184*
Time (minutes) spent on		
Low intensity physical activities	−0.033	−0.024
Moderate intensity physical activities	−0.024	−0.019
Vigorous intensity physical activities	−0.014	0.001

*Significant correlations (*p* < 0.05)

*n* = 2462

Finally, to investigate whether the aforementioned associations are independent ones, we performed a logistic regression to find the predictors of hypertension. The binary logistic regression analysis results revealed that, only age (*p =* 0.001) and weight status (*p* < 0.001) were significantly and independently associated with hypertension. While level of education (*p* = 0.058) was trending towards significance, ethnicity (*p* = 0.783), physical activity level (*p* = 0.684), income status (*p* = 0.440), level of sleeping hours (*p* = 0.348), smoking (*p* = 0.936), consumption of alcohol (*p* = 0.522), fish (*p* = 0.647), meat (*p* = 0.298), dairy (*p* = 0.113), fruits (*p* = 0.594), sweets (*p* = 0.989) or fried snacks (*p* = 0.609) were not significantly associated with hypertension (Table 4). Since the level of education was trending towards significance when it was treated as categorical variable, we further investigated the effect of education on hypertension treating the education as an ordinal (rank) scale variable and results revealed that neither linear (*p* = 0.077) nor quadratic (*p* = 0.051) term was significant (Table 5).

**Table 4:** Variables Associated with Hypertension in the Logistic Regression Model 1

Variable	Chi-Square	*P* value
Age	18.27	0.001*
Ethnicity	0.49	0.783
^#^Level of education	9.14	0.058
Level of smoking	0.39	0.529
Alcohol consumption	0.41	0.522
Physical activity level	0.17	0.684
Level of sleeping	0.88	0.348
Consumption of meat	1.08	0.298
Consumption of fish	0.21	0.647
Consumption of dairy	2.51	0.113
Consumption of fried snacks	0.26	0.609
Consumption of fruits	0.28	0.594
Consumption of sweets	0.0002	0.989
Weight status	19.84	0.000*
Income status	3.75	0.440

^**#**^Level of education entered as a categorical variable**;** * *p* < 0.05

**Table 5:** Odds Ratios of Variables Associated with Hypertension in the Logistic Regression Model 2

Covariate	Hypertension OR (95% CI)	*P* value
Age		0.001
20–30 years Vs. <20 years	0.98 (0.56, 1.72)	
31–40 years Vs. <20 years	1.09 (0.62, 1.91)	
41–50 years Vs. <20 years	1.52 (0.86, 2.71)	
> 50 years Vs. <20 years	2.23 (1.14, 4.35)	
Weight status		0.000
Underweight Vs. Normal weight	0.88 (0.62, 1.26)	
Overweight Vs. Normal weigh	1.34 (1.05, 1.73)	
Obese Vs. Normal weight	1.92 (1.38, 2.67)	
Level of education		
Linear term	19.85 (0.72, 546.16)	0.077
Quadric term	0.34 (0.12, 1.00)	0.051

*n* = 1688

**Fig. 1:**
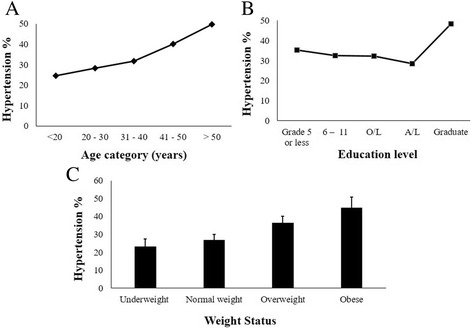
Prevalence of hypertension by age, education level and weight status. Prevalence rates of undiagnosed hypertension (systolic blood pressure ≥ 140 mmHg or diastolic blood pressure ≥ 90 mmHg) according to age categories (**a**), educational status (**b**) and weight status (**c**) are shown. *n* = 2462

Table 6 has summarized the prevalence rates of HT among different groups according age, ethnicity and education level. According to age, the undiagnosed hypertension was highest among the eldest age group (age > 50 years) and the prevalence rate of undiagnosed hypertension increased with age. Participants aged >50 years were 123% (OR: 2.23; 95% CI: 1.14, 4.35) more likely to have hypertension compared to males <20 years of age (Table 5). Further, undiagnosed hypertension was highest among overweight and obese males compared to the normal and underweight males (Fig. 1). The likelihood of being hypertensive was 34.5% (OR: 1.34; 95% CI: 1.05, 1.73) and 92.1% (OR: 1.92; 95% CI: 1.38, 2.67) higher for overweight and obese males, respectively, compared to normal weight males (Table 5).

**Table 6:** Prevalence (95% CI) of unidentified hypertension among males by age, ethnicity, education level, body mass index category and waist circumference level

Variable	Hypertension % (CI)
Age category	
< 20 years	24.5 (20.5, 28.9)
20–30 years	28.2 (25.1, 31.4)
31–40 years	31.8 (28.4, 35.3)
41–50 years	40.1 (35.3, 45.0)
> 50 years	49.6 (41.3, 59.3)
Ethnicity	
Sinhalese	31.7 (29.7, 33.8)
Tamil	29.3 (23.0, 35.9)
Moor	32.3 (25.9, 38.8)
Education level	
No education – grade 5	35.3 (17.6, 59.9)
Grade 6 – grade 11	32.6 (27.8, 37.7)
Ordinary level passed	32.2 (29.6, 34.9)
Advanced level passed	28.5 (25.1, 31.9)
Graduate/postgraduate	48.4 (37.5, 61.9)
Weight status	
Normal weight	27.0 (24.3, 29.9)
Underweight	23. 4 (19.2, 27.6)
Overweight	36.5 (33.1, 40.0)
Obese	44.9 (39.3, 50.8)

*n* = 2462

Among different ethnic groups, Moors showed the highest undiagnosed hypertension prevalence rate (32.3%) while Tamils exhibited the lowest (29.3%). Also, the highest prevalence rate of undiagnosed hypertension was observed in individuals with the highest education level (Table 6).

## Discussion

The aim of the current study was to identify the prevalence rate of undiagnosed HT and factors associated with it in a cohort of males in the Central Province of Sri Lanka. Of the 2462 males studied, 31.7% had undiagnosed HT highlighting the large number of undiagnosed HT likely to be present in this region. Of the studied factors, age and obesity were significantly associated with HT, while level of education showed a trend of association.

In the current study, we excluded subjects who were previously diagnosed with hypertension to eliminate the confounding effects of lifestyle modification following the diagnosis of a non-communicable disease. As a result, all the subjects found to have HT in this study were previously undiagnosed ones. The HT prevalence rate of 31.7% is a higher value compared to previous studies done in Sri Lankan males. A study by Wijewardene et al. during 2000/2001 in four provinces of Sri Lanka showed a mean SBP of 120.1 (SD 20.4) mm Hg and DBP of 75.4 (SD 11.7) mm Hg, with a crude prevalence rate for hypertension (≥ 140/90 mmHg) of 19.4% in males [19]. Mean SBP value of 129.0 ± 19.4 mmHg and DBP value of 75.2 ± 11.6 mmHg for males were reported by Katulanda et al., (2014), in the only national study that has been carried out in Sri Lanka in 2005/2006 [8]. The latter study further reported that the age adjusted prevalence of hypertension was 23.4% among males [8]. Therefore, it is possible that the prevalence of HT in Sri Lanka is on the rise, similar to world trends [1]. Comparatively, in the western Indian population, the overall prevalence of undiagnosed hypertension in 2013 was 26% [20] while it was 36.5% in males and 22.1% in females in USA in 1988–1994 [21]. Taken together, ours and many other studies have highlighted that undiagnosed hypertension is a serious issue which needs to be taken into consideration to overcome the morbidity and mortality associated with hypertension [22–24].

When considering the current study and other previous studies, it is clear that the prevalence rates of hypertension have been increasing over time. One possible reason could be the noticeable increase in obesity and average body mass index in Sri Lankans over the past decades [25], which in turn increased the prevalence of hypertension [26]. Similar trends have been observed in India and China [27, 28]. Further, Danaei, et al. have shown that between 1980 and 2008, SBP has increased 0.8–1.6 mmHg per decade in men and 1.0–2.7 mmHg per decade in women in the South Asian region [29].

The current study shows that increasing age and BMI are significantly associated with HT. We also found trends for level of education to be associated with HT. Previously, Katulanda et al. found that male gender, physical inactivity, diabetes, central obesity and Moor ethnicity were significantly associated with hypertension among Sri Lankans, while no previous studies have looked at dietary patterns in detail [8]. Our data show that until 40 years of age, the odds of being hypertensive is more or less same and the likelihood increases after 40 years. Similar results have also been observed by Shukla, et al. where the prevalence of undiagnosed hypertension in western Indian population was approximately three times higher in the older population (>40 years) than in younger adults [20] . Moreover, many research studies have reported aging as a major risk factor for becoming hypertensive [30, 31]. Mechanistically, the age related risk of HT is attributed to changes in vasculature which includes endothelial dysfunction, thickening of vessel walls, reduced flexibility and arterial stiffening [32].

Obesity is another well-known risk factor for HT [33]. The current study showed that weight, BMI and WC all positively correlated with SBP and DBP. Moreover, the weight status (BMI category) was independently associated with HT in the logistic regression analysis. Further, undiagnosed hypertension was highest among overweight and obese males compared to the normal and underweight males. The likelihood of being hypertensive was 34.5% (OR: 1.34; 95% CI: 1.05, 1.73) and 92.1% (OR: 1.92; 95% CI: 1.38, 2.67) higher for overweight and obese males, respectively, compared to normal weight males. There are several mechanisms underlying the relationship between obesity and HT [33, 34]. One mechanism could be that leptin, an adipokine found in higher levels in obese individuals, acts in the hypothalamus to increase blood pressure through activation of the sympathetic nervous system [34]. We have previously reviewed the role of systemic and adipose tissue renin-angiotensin systems in obesity-related HT [35, 36].

In the current study, level of education was trending towards significance (*p* = 0.058) when it was entered into the model as a categorical variable. According to our findings, HT was high among males who were least educated and the prevalence reduced with the increment of the level of education. Males who had advanced level education (Grade 11–13) showed lowest prevalence. However, when the educational level increased to the top (graduates/postgraduates), the level of HT again increased (Fig. 6). Previous studies have yielded contrasting results on the relationship between educational status and HT. Linear, significant negative associations were reported in some [37, 38], while others have indicated a higher prevalence of HT among the highest educated groups [39].

Fruit and vegetable consumption has a protective effect against hypertension. Alonso et al. showed that consumption of vegetables and fruits has an inverse linear relationship (77% reduction) with the prevalence of undiagnosed hypertension [40]. According to our study, only 14.5% of the study group consumed one or more fruits per day [9]. This overall lower consumption of fruits could be a possible reason why the relationship between fruit intake and HT did not reach significance. Indeed, there are previous studies supporting the current finding of lower daily intake of fruits among Sri Lankans which is well below national recommendations [41]. Mechanistically, the high potassium content in fruits is suggested to lower the blood pressure thus exerting protective effects against HT [42]. Considering the possible relationship between lower fruit intake and the risk of HT as well as the low percentage of adult males meeting the recommended daily fruit intake, it is important to target promotion of fruit intake as an intervention to prevent HT in this area.

Alcohol is also another lifestyle factor which increases the risk of HT [43]. Indeed, some studies have attributed as much as 16% of all cases of HT to alcohol [44]. While 22.5% of the participants in the current study indicated that they were consuming alcohol, it might be an underestimate considering the stigma associated with alcohol intake in this region.

Physical inactivity has been shown to be associated with increased incidence of hypertension in many research studies conducted worldwide including Sri Lanka [8, 45, 46]. In contrast, our findings showed no association between physical activity and HT. It needs to be pointed out that the majority of this study group (64.5%) reported that they engaged in more than 300 min of moderate-intensity physical activity per week. Since the self-reported physical activity levels were obtained, there is a possibility of over-reporting their physical activity levels. Indeed, it is well known that individuals over report physical activity levels [47]. This factor needs to be taken into account when interpreting the current finding of no association between physical activity level and HT in the current study.

Of the other factors considered in this study, we did not find an association between the number of sleep hours and HT. Previous studies have shown conflicting results regarding the association between sleep hours and blood pressure. Some have shown that short sleep duration is associated with HT [48], while others have shown that this association is only seen in women [49]. Since the current study group only comprised males, it is certainly warrented to further explore this relationship in Sri Lankans, especially in women.

While our cross-sectional study provided useful findings on the prevalence of undiagnosed hypertension and underlying risk factors among adult males of the Central province of Sri Lanka, there are some limitations. Since >95% of the individuals who presented for this routine medical examination were males, we only included males for this study. Further, we could not investigate the relation between salt intake and HT.

## Conclusion

The high prevalence rate of undiagnosed hypertension seen in working age males signifies a major health problem which requires urgent steps to be taken for its prevention and control. Routine screening is important to identify these undiagnosed individuals with HT, especially with advancing age. Since obesity was identified as a factor associated with HT, interventions targeted at reducing obesity rates are needed to curb the likely increases in cardiovascular morbidity and mortality of HT in this region.

## Abbreviations

BMI: Body mass index
CI: Confidence interval
DBP: Diastolic blood pressure
HT: Hypertension
IPAQ: International physical activity questionnaire
SBP: Systolic blood pressure
SD: Standard deviation
WC: Waist circumference
WHO: World health organization
